# Seven Year Old Male with Tricuspid Endocarditis 

**DOI:** 10.24908/pocus.v6i1.14755

**Published:** 2021-04-22

**Authors:** Omar Damji, Russ A Lam, Mark Bromley, Melanie Willimann

**Affiliations:** 1 Department of Emergency Medicine, University of Calgary Cumming School of Medicine Calgary, Alberta; 2 Department of Pediatric Emergency Medicine, University of Calgary Cumming School of Medicine Calgary, Alberta; 3 Department of Pediatrics, University of Calgary Cumming School of Medicine Calgary, Alberta

**Keywords:** POCUS, pediatric endocarditis, emergency medicine

## Abstract

Pediatric endocarditis, a rare entity in developed countries, remains a challenging diagnosis to make in children. We present an uncommon etiology of shortness of breath on exertion (SOBOE) in a 7-year-old male presenting with two weeks of nocturnal fever, malaise and fatigue following a viral prodrome. Point of care ultrasound (POCUS) led to suspicion for a ventricular septal defect (VSD) with tricuspid valve (TV) endocarditis, which was ultimately confirmed by formal echocardiography. This ultrasound diagnosis allowed emergency clinicians to order blood cultures under the suspicion of endocarditis as well as expedited antibiotic treatment.

## Case

Pediatric endocarditis, is a rare diagnosis in developed countries 1. Children from third world countries carry a unique set of risk factors that may predispose them to this rare and invasive infection with potential for severe sequalae 1. Poor dentition, presence of a congenital cardiac abnormality (pre and post repair), foreign body, and being immunocompromised all carry independent risk for pediatric endocarditis 1. This is a case of a previously healthy 7-year-old male who moved from Somalia to Canada 1 year prior to presentation in the ED. He took no medications, had no allergies, and his immunizations were up to date. He presented with a 2-week history of nocturnal fever, shortness of breath on exertion (SOBOE), malaise and fatigue. His mother described fever beginning at 1800 each night and persisting over the course of the evening. The fever did not respond to antipyretic treatment and would break in the morning. Cough and coryza preceded his nocturnal fevers. Over the 3 days prior to his presentation, he developed significant SOBOE to the point of dyspnea while drinking water or taking a few steps. His mother described him as fatigued but interactive. His oral intake was still maintained and he had no history of bowel or bladder symptoms. On review of systems, there was no rash, toxic ingestion, or trauma. He had no history of similar symptoms previously. He was seen in the community by a paediatrician and sent in with the presumptive diagnosis of myocarditis. 

**Figure 1:**
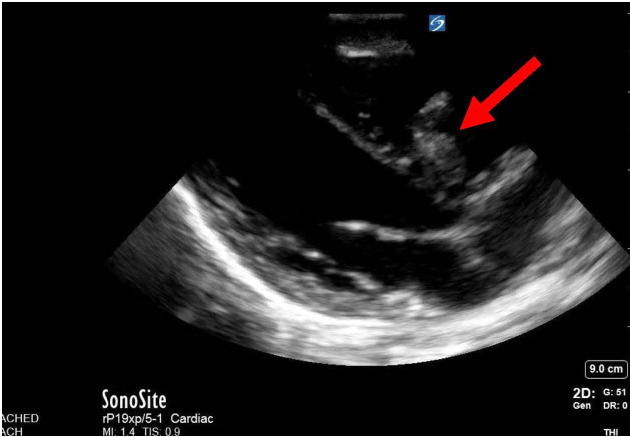
This is a still image of a parasternal long view of the heart. Overall, there are some irregularities in the basal aspect of the inter-ventricular septum and thickness to one of the leaflets of the tricuspid valve.

**Figure 2:**
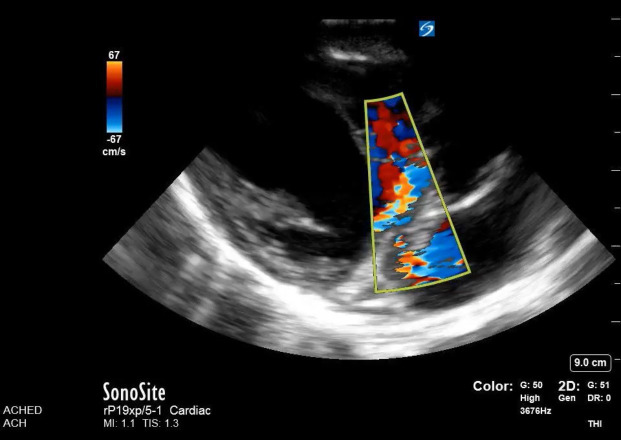
This is a still image of a parasternal long view of the heart. Overall, there is a jet indicating regurgitant flow across the basal aspect of the inter-ventricular septum. There seems to be doppler flow in the left atrium. On an additional view of the mitral valve, there was no evidence of mitral regurgitation. This jet extended within the left atrium may be a reflection of added gain from the jet of the VSD. With the clinical exam of a holosystolic murmur at the left lower sternal boarder, and this image, a ventricular septal defect (VSD) was suspected.

**Figure 3:**
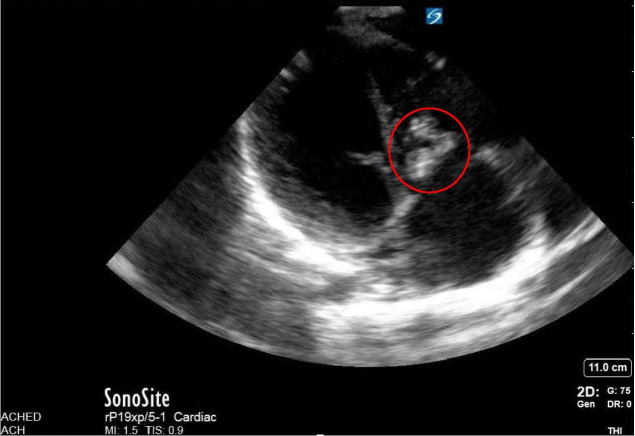
This is a still image of an apical four chamber view of the heart. Overall, there is a thickness noted in one of the leaflets of the tricuspid valve. With his history of fever, suspicion of tricuspid valve endocarditis leading to symptoms of right heart failure was suspected.

Upon arrival to the Alberta Children’s Hospital Emergency Department, he had a temperature of 37.9 for which he received an antipyretic at triage, heart rate of 118, blood pressure 94/45, respiratory rate of 35 and was saturating 94% on room air. On exam, he had an elevated JVP to the level of the tragus, grade III/VI holosystolic murmur at his left lower sternal border, hepatomegaly with his liver edge 5cm below the costal margin. His abdominal exam was otherwise benign. He had decreased breath sounds bilaterally, but no adventitia. He did not have stigmata of endocarditis. Point of care ultrasound (POCUS) showed normal contractility, normal left ventricular size, regurgitant flow at the basal aspect of the septum (Figure 1 and Figure 2/Video S1 and Video S2), a large mobile irregularly shaped mass at one leaflet of the tricuspid valve (TV) with evidence of regurgitant flow in the apical four chamber view (Figure 1, Figure 3/Video S3 and Video S4] as well as with doppler in the IVC indicating a basal ventricular septal defect (VSD – left to right shunt), TV endocarditis and evidence of tricuspid shrouding/tricuspid regurgitation at the level of the valve. There was abnormal regurgitant doppler flow at the inferior vena cava. 

Given the clinical history, presentation, physical exam and bedside ultrasound findings, the diagnosis of a TV endocarditis in the context of an undiagnosed basal VSD was entertained in the context of a preceding viral prodrome and poor dentition. This diagnosis was confirmed on formal echocardiography and blood cultures were positive for abiotrophia defectivia 2. This bacteria is a variant of the viridans species of streptococcus, commonly known as the causative bug for endocarditis 2. It is quite virulent and has a predisposition for affecting endovascular structures including heart valves 2. It is also a common agent involved in culture negative endocarditis 2. He received a total of 6 weeks of antibiotics prior to cardiovascular surgery. He underwent perimembranous VSD closure and tricuspid valve reconstruction. Post operatively, he was placed on an additional four weeks of antibiotics. Unfortunately, he also developed post-operative pericarditis confirmed by friction rub and diffuse ST segment elevation on his electrocardiogram.

## Cardiac Ultrasound in Pediatric Emergency Departments

Bedside ultrasonography has become increasingly available across many adult and pediatric emergency departments 3. It is utilized for many indications including: acute trauma, abdominal, cardiac, musculoskeletal and infectious presentations as well as for regional anesthesia, confirming fracture reduction, appraising ocular concerns, landmarking for central venous access. Point of care ultrasound is well described in adult literature. Applications to paediatrics remains simple and few. Hesitation regarding the use of bedside ultrasound in paediatrics remains strong in light of a rather small and slowly developing pool of literature 3. In recent years, many centres across the world have recognized this gap and attempted to validate the use of bedside ultrasound in the appraisal of acute pediatric presentations within the emergency department 3, 4, 5, 6. A myriad of adult studies suggest skill acquisition over a short training module is possible for assessing left ventricular (LV) function and presence of pericardial effusion 7, 8. When appropriate training occurs, point of care cardiac ultrasound (POCUS) can be an accurate and fast way to make clinical decisions. Agreement between the trainee learning cardiac ultrasound and the cardiologist after a training session was seen in 93**%** of cases for visual estimated of global cardiac function, and 98% for detection of pericardial effusion 3. For pericardial effusion, sensitivity of 60%, specificity of 100%, positive predictive value of 100% and negative predictive value of 97.9% is quoted 9. The sensitivity of cardiac POCUS in pediatric emergency physicians in detecting LV dysfunction is quoted as 95% and a specificity of 83% 3. If one is well trained in cardiac POCUS, the results will be in line with a formal echo and can expedite care for patients 10. Overall, adult and pediatric studies suggest bedside cardiac assessments are feasible, guide management, and inform disposition effectively 10. 

## Conclusion

Pediatric endocarditis, a rare entity in developed countries, remains a challenging diagnosis to make in children 11. Point of care cardiac ultrasound can provide diagnostic information to increase comfort with clinical appraisal, management, and disposition. Without ultrasound, this child would still have been admitted and had undergone a formal echocardiogram. However, with the power of bedside ultrasound, we were able to facilitate early appraisal for endocarditis and empiric antibiotics. This is the first report we are aware of describing the diagnosis of a VSD and tricuspid valve endocarditis using POCUS from the pediatric emergency department. 

## Disclosures

The authors have no conflicts of interest to declare.

## Supplementary Material 

Video S1 Parasternal long view of the heart

Video S2 Parasternal long view of the heart with Doppler.

Video S3 Apical four chamber view of the heart.

Video S4 Apical four chamber view of the heart with Doppler.
